# Tofacitinib overcomes an IFNγ‐induced decrease in NK cell‐mediated cytotoxicity via the regulation of immune‐related molecules in LC‐2/ad

**DOI:** 10.1111/1759-7714.13847

**Published:** 2021-01-24

**Authors:** Riki Okita, Katsuhiko Shimizu, Yuji Nojima, Shinsuke Saisho, Masao Nakata

**Affiliations:** ^1^ Department of General Thoracic Surgery Kawasaki Medical School Kurashiki Japan; ^2^ Division of Thoracic Surgery National Hospital Organization Yamaguchi Ube Medical Center Ube Japan

**Keywords:** IFNγ, JAK‐STAT pathway, NK cell, nonsmall cell lung cancer (NSCLC), tofacitinib

## Abstract

**Background:**

Immune checkpoint inhibitors targeting the programmed cell death‐1 (PD‐1)/PD‐1 ligand 1 (PD‐L1) axis have shown promising results in patients with nonsmall cell lung cancer (NSCLC). One major PD‐L1 inducer is IFNγ, which is secreted by T cells and NK cells. Importantly, IFNγ‐induced PD‐L1 is one of the major mechanisms by which cancer cells escape host immunity.

**Methods:**

Here, we found that the NSCLC cell line, LC‐2/ad, has a unique character; the PD‐L1 expression in these cells is up‐regulated by both IFNγ and epidermal growth factor (EGF).

**Results:**

Comparative analysis of the cell signaling pathway showed that IFNγ activates STAT1 signaling, while EGF activates AKT, MAPK, and ribosomal protein S6 kinase in LC‐2/ad cells. IFNγ‐induced PD‐L1, but not EGF‐induced PD‐L1, was clearly blocked by the JAK‐STAT inhibitor tofacitinib. Interestingly, IFNγ decreased the expression of NK cell‐activating ligands while increasing the expression of MHC class I molecules, resulting in a phenotype that can easily escape from NK cells, theoretically. Finally, we showed that IFNγ stimuli attenuated NK cell‐mediated cytotoxicity in LC‐2/ad cells, which was, however, blocked by tofacitinib.

**Conclusions:**

Taken together, our study shows that tofacitinib blocks the IFNγ‐induced transformation from an NK cell‐sensitive phenotype to an NK cell‐resistant one in IFNγ‐reacted LC‐2/ad cells, thereby implicating that tofacitinib may be a promising agent to overcome IFNγ‐induced tumor immune escape, although it may be adapted to the limited number of NSCLC patients.

## Graphical abstract

The JAK‐STAT inhibitor tofacitinib blocks the IFNγ‐induced transformation from an NK cell‐sensitive phenotype to an NK cell‐resistant one in IFNγ‐reacted LC‐2/ad cells, thereby implicating that tofacitinib may be a promising agent to overcome IFNγ‐induced tumor immune escape, although it could be adapted to the limited number of NSCLC patients.

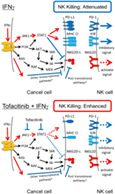

## INTRODUCTION

Lung cancer is the leading cause of cancer‐related deaths worldwide.^1^ Clinical studies have established immune checkpoint inhibitors targeting the programmed cell death‐1 (PD‐1)/PD‐1 ligand 1 (PD‐L1) axis as standard therapeutic regimens for patients with nonsmall cell lung cancer (NSCLC); however, around 70% patients have no objective response to PD‐1/PD‐L1 checkpoint blockade therapy.^2^, ^3^ Therefore, it is important to develop strategies to overcome the drug‐resistant mechanism of PD‐1/PD‐L1 blockade. The combination of PD‐1/PD‐L1 targeted therapy with other types of immunotherapy, such as cytotoxic T‐lymphocyte associated protein‐4‐targeting drugs^4^ and chimeric antigen receptor T cell therapy,^5^ has acquired renewed interest.

Cancer immunotherapy induces the activation of immune effector cells, such as NK cells or T cells.^6^, ^7^ Activated NK cells and T cells secrete IFNγ, and exposure to IFNγ leads to PD‐L1 overexpression in cancer cells,^8^ resulting in tumor escape from host immunity. That means blocking IFNγ‐induced overexpression of PD‐L1 in cancer cells theoretically prolongs the effect of immunotherapy. It is also of particular interest to investigate the effect of IFNγ on the expression of other immune checkpoint molecules.

In this study, we show that the JAK‐STAT inhibitor tofacitinib can block LC‐2/ad cells, thereby changing their characteristic from an NK cell‐resistant phenotype to NK cell‐sensitive phenotype via the inhibition of IFNγ‐induced reaction, resulting in an enhanced NK cell‐mediated cytotoxicity against IFNγ‐reacted LC‐2/ad cells.

## MATERIALS AND METHODS

### Cell culture and reagents

The human NSCLC cell lines LC‐2/ad, A549, RERF‐LC‐AI, and RERF‐LC‐KJ were obtained from Riken BRC through the National Bio‐Resource Project of the MEXT (Tsukuba), while PC‐9 was obtained from the IBL cell bank (Gunma). The genotypes of all cell lines were identified using the PowerPlex 16 STR system (Promega). The cell lines were maintained as previously described.^9^ For cell culture, tofacitinib (#S5001, Selleck), gefitinib (#13166; Cayman), LY294002 (#70920; Cayman), PD98059 (#10006726; Cayman), and PF4708671 (#4032; Tocris) stock solutions were prepared in DMSO (Sigma‐Aldrich), whereas recombinant human IFNγ (#11500; PBL Assay Science) and epidermal growth factor (EGF) (#236‐EG; R&D Systems) stock solutions were prepared in PBS (−).

### Flow cytometry

Extracellular staining was performed using fluorochrome‐conjugated antibodies as previously described.^10^ The following antibodies were used for staining: PE‐labeled major histocompatibility complex class I chain A and B (MICA/B) (clone 6D4; BioLegend), allophycocyanin‐labeled UL16 binding protein (ULBP)‐2/5/6 (clone 165 903; R&D Systems), PE‐labeled PD‐L1 (clone 29E.2A3; BioLegend), allophycocyanin‐labeled HLA‐A, B, and C (clone G46‐2.6; BioLegend), as well as PE‐ or allophycocyanin‐labeled anti‐mouse IgG1κ (clone MOPC‐21; BioLegend) and IgG2bκ (clone MOPC‐173; BioLegend) as isotype controls. The cells were assayed using a FACSCanto II flow cytometer (BD Biosciences) and analyzed using FlowJo software 6.4.7 (Treestar Ashland). The increase in mean fluorescence intensity (ΔMFI) was calculated as (MFI with specific mAb – MFI with isotype control)/MFI with isotype control. The relative MFI (rMFI) values were calculated to compare the differences between ΔMFI values of a specific treatment and control as 100 × (ΔMFI of a specific treatment/ΔMFI of the control treatment).

### Receptor tyrosine kinase Ab array

LC‐2/ad cells were cultured in six‐well plates to 80% confluence followed by treatment of cells with 0.5 ng/mL IFNγ or 100 ng/mL EGF for 1 h. After medium removal, cells were rinsed with ice‐cold PBS (−) and lysed with the lysis buffer available in the PathScan receptor tyrosine kinase (RTK) Ab array kit (#7982; Cell Signaling Technology). The protein concentration was measured using the BCA protein assay kit (#T9300A; Takara Bio) according to the manufacturer's instructions. Equal amounts of proteins (60 μg/well) from LC‐2/ad cells were incubated with RTK Ab array according to the manufacturer's protocol. The array spot signals were acquired using the Odyssey Infrared Imaging System (LI‐COR Biosciences; Lincoln).

### NK cell‐mediated cytotoxicity assay

Peripheral blood mononuclear cells were collected from healthy donors by gradient centrifugation in Vacutainer cell preparation tubes (BD Biosciences). NK cells were negatively purified using an NK cell isolation kit (Stem Cell Technologies) and incubated with 100 IU/mL IL‐2 (Teceleukin) for 24 h as previously described.^9^ The LC‐2/ad cells pretreated with or without 1 μM tofacitinib followed by 0.5 ng/mL IFNγ were tested for sensitivity to NK cell‐mediated cytotoxicity using a lactate dehydrogenase (LDH) release assay kit (CytoTox 96 Non‐Radioactive Cytotoxicity Assay; Promega) and a Varioskan Flash Spectral Scanning Multimode Reader (Thermo Scientific) as previously described.^9^ For the LDH release assay, blood samples were collected only from researchers who were involved with this study, therefore written informed consent was not required. The ethical research committee of Kawasaki Medical School approved both the study and this procedure (No. 1217‐5).

### Apoptosis assay

The induction rate of apoptotic NK cells was determined by flow cytometry on Annexin V staining with allophycocyanin‐labeled Annexin V (#640920; BioLegend) according to the manufacturer's instructions. The percentage of apoptotic NK cells was calculated.

### Statistics

Differences in means were evaluated using Student's *t*‐test. All analyses were performed at a significance level of 5% (*p* < 0.05) using GraphPad Prism 5 (GraphPad Software Inc.).

## RESULTS

### Both IFNγ and EGF enhance the expression of PD‐L1 in LC‐2/ad cells but through different signaling pathways

**FIGURE 1:**
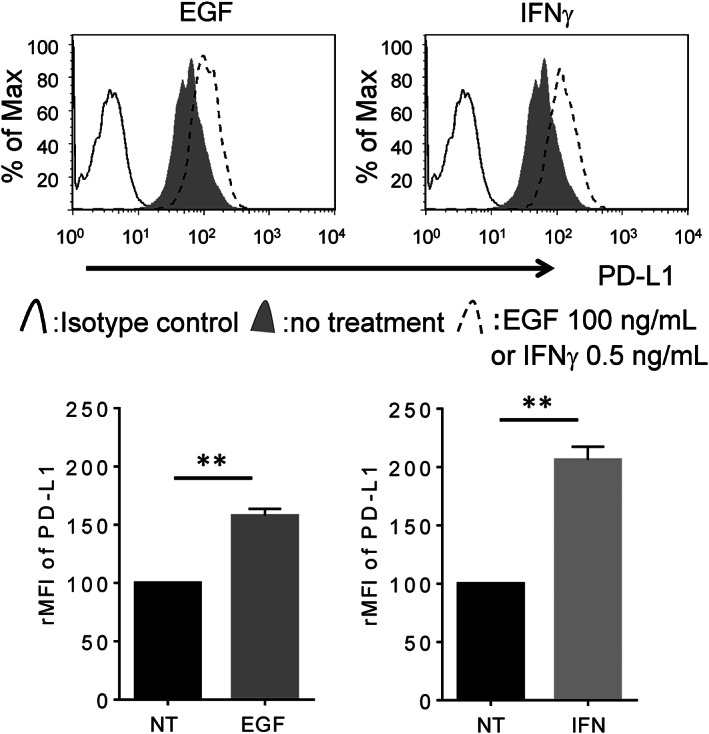
PD‐L1 overexpression is induced by both EGF and IFNγ in LC‐2/ad cells. LC‐2/ad cells were treated with 100 ng/mL EGF or 0.5 ng/mL IFNγ for 24 h followed by evaluation of PD‐L1 expression using flow cytometry. The results represent three independent experiments. The relative mean fluorescence intensity (rMFI) of PD‐L1 was calculated based on three independent experiments and evaluated using Student's *t*‐test. Bars indicate SD. ^**^ *p* < 0.01. NT, no treatment control

We previously reported that IFNγ enhanced PD‐L1 expression in the A549 lung adenocarcinoma cell line, while EGF enhanced PD‐L1 in the PC‐9 lung adenocarcinoma cell line, but the opposite is not true.^9^, ^11^, ^12^ Interestingly, in the current study, we found that PD‐L1 is up‐regulated by both IFNγ and EGF in only LC‐2/ad cells (Figures 1, S1 and S2), which led us to focus on the regulation mechanism of PD‐L1 expression in LC‐2/ad cells in the subsequent experiments. It was reported that IFNγ enhances PD‐L1 via the JAK–STAT pathway,^13^ while epidermal growth factor receptor (EGFR) signaling regulates PD‐L1 expression via the PI3K‐AKT pathway.^14^ Next, we evaluated the signaling pathway that regulates IFNγ‐ or EGF‐induced PD‐L1 expression. RTK Ab array results showed that IFNγ activated the STAT1 pathway, while EGF activated AKT, p44/22 MAPK, and ribosomal protein S6 kinase (S6K) in LC‐2/ad cells (Figure S3).

### JAK‐STAT inhibitor, tofacitinib, blocks IFNγ‐induced PD‐L1 overexpression but not EGF‐induced PD‐L1 overexpression in LC‐2/ad cells

**FIGURE 2:**
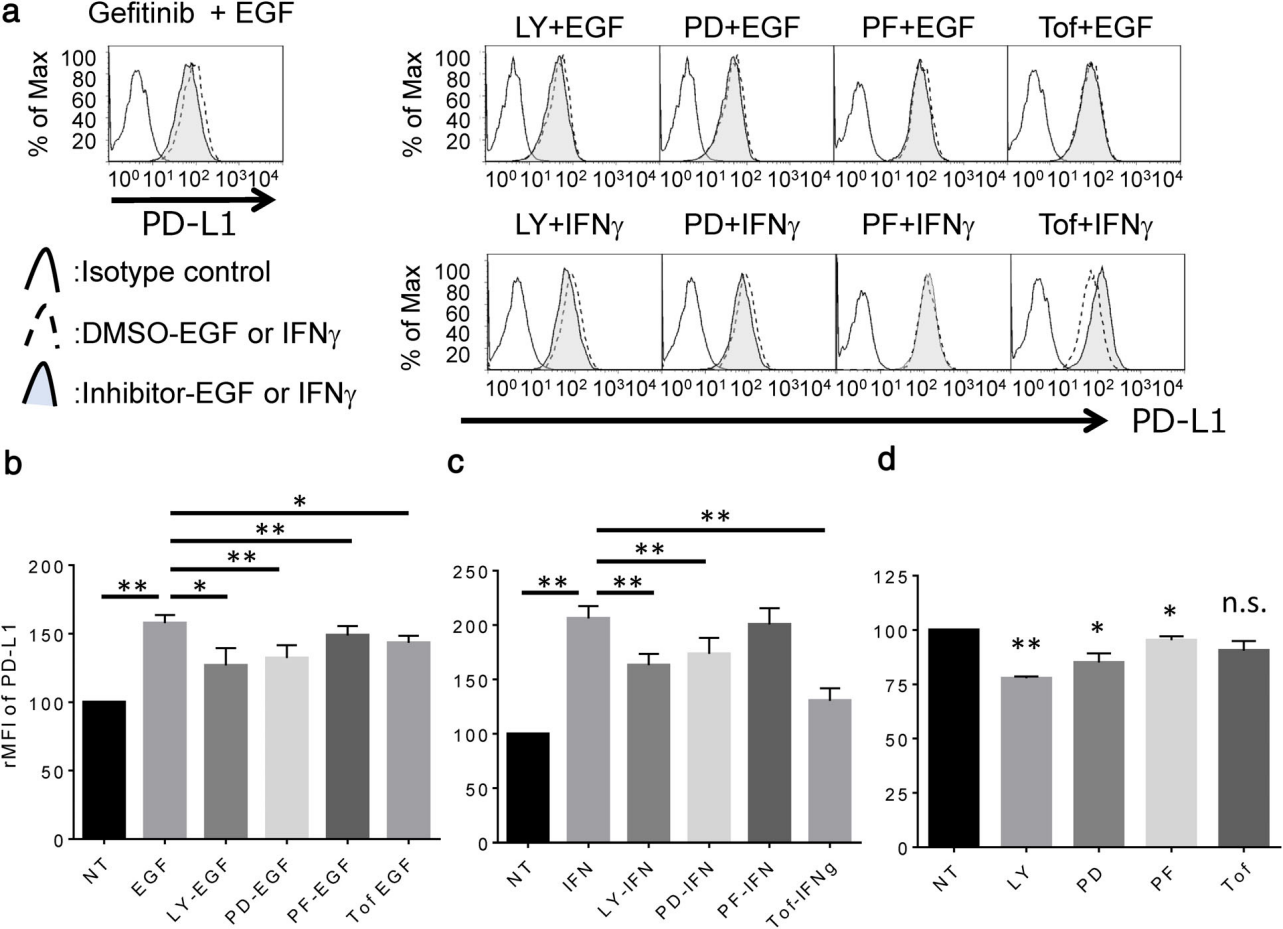
Expression of PD‐L1 in LC‐2/ad cells treated with IFNγ or EGF followed by each signal inhibitor. (a) The expression level of PD‐L1 in LC‐2/ad cells pretreated with each signaling inhibitor (gefitinib 1 μM; LY, LY294002 10 μM; PD, PD98059 10 μM; PF, PF4708671 10 μM; Tof, tofacitinib 1 μM) for 2 h followed by 100 ng/mL EGF or 0.5 ng/mL IFNγ for 24 h evaluated by flow cytometry. The results represent three independent experiments. The relative mean fluorescence intensity (rMFI) of PD‐L1 was calculated in cells pretreated with each signal inhibitor followed by (b) IFNγ and followed by (c) EGF, or (d) treated with each signal inhibitor alone based on three independent experiments and evaluated using Student's *t*‐test. Bars indicate SD. ^*^ *p* < 0.05, ^**^ *p* < 0.01, n.s., no significance; NT, no treatment control

Next, LC‐2/ad cells were treated with several signal inhibitors targeting the candidate pathway to assess the blocking effect on EGF‐ or IFNγ‐induced PD‐L1 up‐regulation. EGF‐induced PD‐L1 expression is blocked by the EGFR tyrosine kinase inhibitor, gefitinib, or signal inhibitor targeting PI3K (LY294002), MEK1 (PD98059), or S6K (PF4708671), which is the downstream pathway of EGFR as well as the JAK‐STAT inhibitor, tofacitinib (Figure 2a,b). However, IFNγ‐induced PD‐L1 expression is significantly blocked by tofacitinib, while the expression is relatively less but significantly affected by LY294002 and PD98059 in LC‐2/ad cells (Figure 2a,c). Basal expression of PD‐L1 is down‐regulated by LY294002, PD98059, and PF4708671, but no change is observed with tofacitinib (Figure 2d). These findings suggest that EGF‐induced PD‐L1 expression is mainly regulated by the downstream pathway of EGFR via PI3K, MEK1, and S6K, while IFNγ‐induced PD‐L1 expression is mainly regulated by the JAK–STAT pathway in LC‐2/ad cells. Interestingly, basal expression of PD‐L1 is regulated by PI3K, MEK1, and S6K signaling pathways, which are the main downstream pathways of EGFR. Since LC‐2/ad cells have the EGFR driver mutation, L858R,^15^ it makes sense that the downstream signaling pathway of EGFR is constitutively activated in LC‐2/ad cells.

### IFNγ up‐regulates MHC class I molecules via the JAK‐STAT pathway in LC‐2/ad cells

**FIGURE 3:**
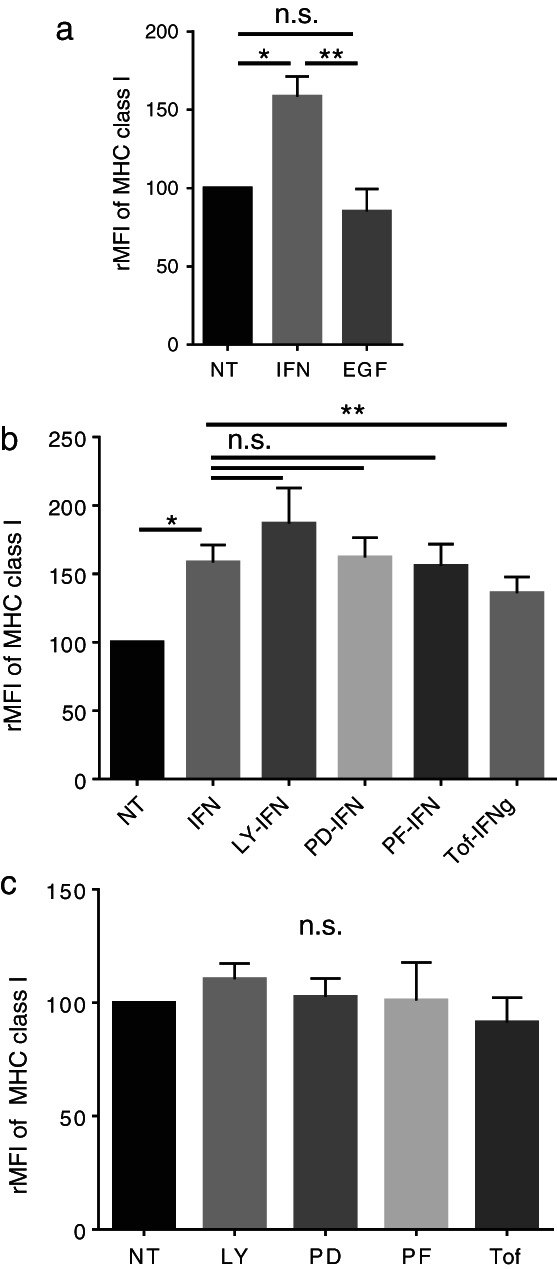
IFNγ increases the expression of MHC class I molecules via the JAK‐STAT pathway, which is blocked by tofacitinib in LC‐2/ad cells. LC‐2/ad cells were (a) treated with 100 ng/mL EGF or 0.5 ng/mL IFNγ for 24 h, (b) pretreated with each signaling inhibitor for 2 h followed by 0.5 ng/mL IFNγ for 24 h, or (c) treated with each signaling inhibitor alone and evaluated by flow cytometry. The relative mean fluorescence intensity (rMFI) of MHC class I molecules was calculated based on three independent experiments and evaluated using Student's *t*‐test. Bars indicate SD. ^*^ *p* < 0.05, ^**^ *p* < 0.01, n.s., no significance; NT, no treatment control; IFN, IFNγ; LY, LY294002 (10 μM); PD, PD98059 (10 μM); PF, PF4708671 (10 μM); Tof, tofacitinib (1 μM)

Previous reports have shown that IFNγ increases the expression of MHC class I molecules in tumor cells,^16^ while EGF decreases it.^17^ In line with the previous reports, IFNγ was found to up‐regulate the expression of MHC class I molecules in LC‐2/ad cells (Figure 3a). Moreover, tofacitinib significantly blocks IFNγ‐induced increase in MHC class I molecules, HLA‐A, B, C (Figure 3b). Interestingly, other signal inhibitors cannot block IFNγ‐induced MHC class I molecules (Figure 3b), suggesting that IFNγ‐induced increase in MHC class I expression is mainly regulated via JAK‐STAT signaling. In contrast, EGF has no effect on the expression of MHC class I molecules in LC‐2/ad cells (Figure 3a). Moreover, the basal expression of MHC class I molecules is not regulated via the PI3K, MEK1, S6K, or JAK‐STAT pathways (Figure 3c). These findings suggest that both EGF and IFNγ enhance the expression of PD‐L1, but they show different effects on MHC class I expression in LC‐2/ad cells. The MHC class I molecules are immune checkpoint molecules recognized by killer cell immunoglobulin‐like receptor (KIR) on NK cells, resulting in inactivation of NK cells.^18^, ^19^ Therefore, IFNγ‐induced increase in the expression of MHC class I molecules is a suitable strategy for cancer cells to escape from NK cells.

### IFNγ decreases NKG2D ligands, MICA/B and ULBP2/5/6, in LC‐2/ad cells, while EGF increases MICA/B but decreases ULBP2/5/6

**FIGURE 4:**
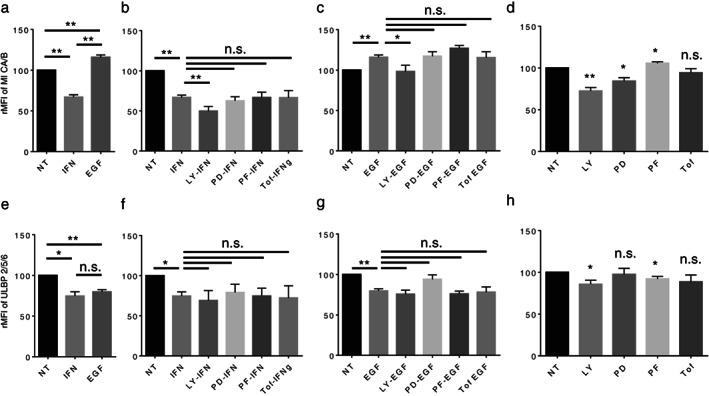
IFNγ decreases the expression of NKG2D ligands MICA/B and ULBP2/5/6 in LC‐2/ad cells. The expression of MICA/B in LC‐2/ad cells (a) treated with 100 ng/mL EGF or 0.5 ng/mL IFNγ for 24 h, (b) pretreated with each signaling inhibitor for 2 h followed by 0.5 ng/mL IFNγ for 24 h, (c) followed by 100 ng/mL EGF for 24 h, or (d) treated with each signal inhibitor alone and evaluated by flow cytometry. The expression of ULBP2/5/6 in LC‐2/ad cells (e) treated with 100 ng/mL EGF or 0.5 ng/mL IFNγ for 24 h, (f) pretreated with each signaling inhibitor for 2 h followed by 0.5 ng/mL IFNγ for 24 h, (g) followed by 100 ng/mL EGF for 24 h, or (h) treated with each signal inhibitor alone and evaluated by flow cytometry. The relative mean fluorescence intensity (rMFI) of MICA/B or ULBP2/5/6 was calculated based on three independent experiments and evaluated using Student's *t*‐test. Bars indicate SD. ^*^ *p* < 0.05, ^**^ *p* < 0.01, n.s., no significance; NT, no treatment control; IFN, IFNγ; LY, LY294002 (10 μM); PD, PD98059 (10 μM); PF, PF4708671 (10 μM); Tof, tofacitinib (1 μM)

It was previously reported that IFNγ decreases the expression of NK group 2 member D (NKG2D) ligands in cancer cells.^20^ Moreover, we previously showed that IFNγ stimuli decreased the expression of NKG2D ligands in the NSCLC cell lines A549 and PC‐9.^12^ In line with our previous work, IFNγ was found to down‐regulate the expression of the NKG2D ligand MICA/B in LC‐2/ad cells (Figure 4a). Interestingly, tofacitinib as well as other signal inhibitors do not block IFNγ‐induced down‐regulation of MICA/B in this cell line (Figure 4b), suggesting that an unknown pathway regulates IFNγ‐induced down‐regulation of MICA/B. Interestingly, LY294002 shows an additional reducing effect on IFNγ‐induced MICA/B down‐regulation, suggesting that the regulation mechanisms between IFNγ‐induced and LY294002‐induced MICA/B down‐regulation are independent. EGF induced MICA/B up‐regulation, which was blocked by LY294002 (Figure 4c), suggesting that EGF‐induced MICA/B up‐regulation is mainly regulated by EGFR signaling via the PI3K pathway. Basal expression of MICA/B is down‐regulated by LY294002 or PD98059, but not by tofacitinib (Figure 4d), suggesting that the basal expression of MICA/B is not regulated via the JAK‐STAT pathway in LC‐2/ad cells, but by EGFR signaling via PI3K and MEK1.

We assessed the other important NKG2D ligand, ULBP2/5/6, in these cells. Both IFNγ and EGF decrease ULBP2/5/6 expression (Figure 4e). Both IFNγ‐ and EGF‐induced decrease in ULBP2/5/6 was not blocked by tofacitinib as well as other signal inhibitors (Figure 4f,g), suggesting that stimuli‐induced decrease in ULBP2/5/6 is regulated by an unknown pathway. The basal expression of ULBP2/5/6 is down‐regulated by LY294002 and PF4708671, but not by PD98059 or tofacitinib (Figure 4h). In summary, IFNγ decreases the expression of NKG2D ligands, and this transformation is also a reasonable immune escape mechanism.

### IFNγ reduces NK cell‐mediated cytotoxicity in LC‐2/ad cells, and tofacitinib overcomes it without induction of apoptosis in NK cells

**FIGURE 5:**
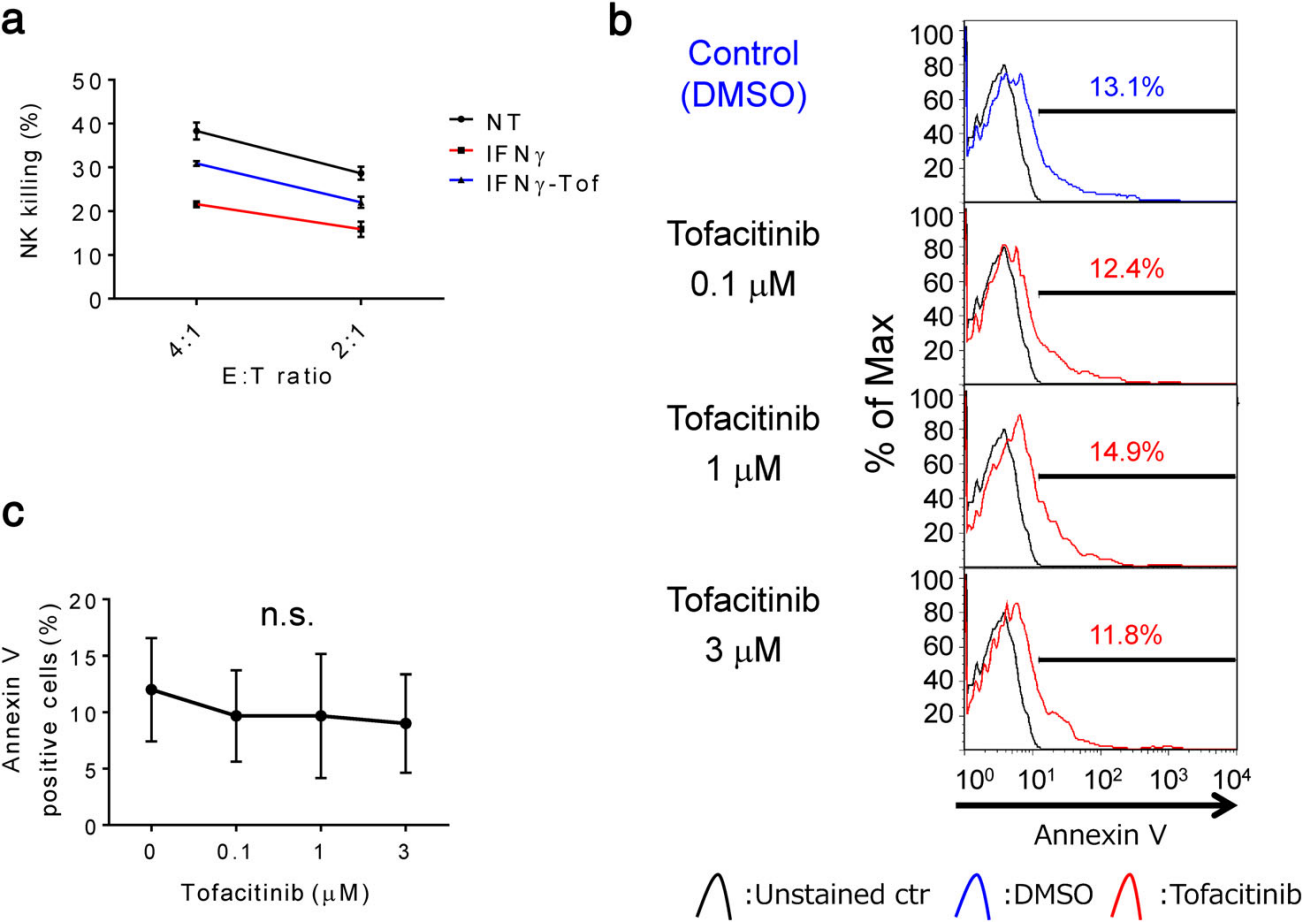
Tofacitinib overcomes IFNγ‐induced decrease in NK cell‐mediated cytotoxicity in LC‐2/ad cells. (a) LC‐2/ad cells treated with 0.5 ng/mL IFNγ for 24 h, LC‐2/ad cells pretreated with 1 μM tofacitinib for 2 h followed by 0.5 ng/mL IFNγ for 24 h, and untreated LC‐2/ad cells were subjected to LDH release assay for 4 h using IL‐2‐activated NK cells as effector cells. Data are presented as the means of triplicate experiments. The results represent three independent examinations. (b) The panels show representative images of the apoptotic assay with Annexin V in NK cells. The IL‐2‐activated NK cells were treated with several concentrations of tofacitinib for 24 h, followed by assessment of Annexin V positive apoptotic cells by flow cytometry. (c) The proportion of Annexin V positive apoptotic cells was calculated based on three independent experiments and evaluated using Student's *t*‐test. Bars indicate SD. n.s., no significance; NT, no treatment control; Tof, tofacitinib

Theoretically, IFNγ‐induced increase in PD‐L1 and MHC class I expression as well as IFNγ‐induced decrease in NKG2D ligand expression in cancer cells attenuate NK cell‐mediated cytotoxicity. To evaluate the effect of tofacitinib on the sensitivity to NK cell‐mediated death, LC‐2/ad cells were pretreated with tofacitinib for 2 h followed by IFNγ for 24 h prior to the assay. IFNγ attenuated NK cell‐mediated cytotoxicity in LC‐2/ad cells and, as expected, tofacitinib pretreatment blocked IFNγ‐induced decrease in NK cytotoxicity in LC‐2/ad cells (Figure 5a). These findings indicate that tofacitinib is a promising agent that can be used to overcome the IFNγ‐induced cancer immunoescape mechanism. We were concerned about the immunosuppressive effect of tofacitinib on NK cells since tofacitinib is an antirheumatic drug^21^ that inactivates the function of T cells.^22^ Interestingly, less than 3 μM of tofacitinib does not show an apoptotic effect on NK cells (Figure 5b,c), suggesting that tofacitinib does not suppress but enhances NK cell‐mediated cytotoxicity against IFNγ‐reacted LC‐2/ad cells. Hence, the IFNγ‐induced immune escape mechanisms in LC‐2/ad cells are schematically summarized in Figure S4.

## DISCUSSION

In this study, we showed that both EGF and IFNγ increase PD‐L1 expression with different mechanisms. Moreover, IFNγ‐induced PD‐L1 expression is blocked by tofacitinib, which is a JAK‐STAT inhibitor broadly used in the treatment of rheumatoid arthritis.^21^ Interestingly, IFNγ has been shown to up‐regulate the NK cell‐inhibitory ligand, MHC class I molecule, in LC‐2/ad cells. In addition, the down‐regulation of NKG2D ligands in tumor cells induced by IFNγ alters the tumor cell phenotype from NK cell‐sensitive to NK cell‐resistant. Additionally, IFNγ‐induced up‐regulation of MHC class I molecules was blocked by tofacitinib, while IFNγ‐induced down‐regulation of NKG2D ligands was not.

It is well known that the expression of PD‐L1 is primarily up‐regulated by IFNγ‐induced activation of the JAK‐STAT pathway^13^ and by EGFR signaling^23^, ^24^ via the PI3K‐AKT pathway.^14^ Conversely, the expression of MHC class I molecules is up‐regulated by several stimuli including IFNγ^16^ and down‐regulated through the EGFR signaling pathway^17^ and HER2.^25^ Expression of NKG2D ligands is primarily regulated by the ataxia‐telangiectasia‐mutated, ataxia‐telangiectasia‐mutated, and Rad‐3‐related protein kinase pathways^26^ and is also up‐regulated by HER3 or EGFR signaling via the downstream pathway of PI3K/AKT.^9^, ^10^, ^27^ Moreover, IFNγ decreases the expression of NKG2D ligands not only at the transcriptional level via STAT1 signaling^20^ but also at the post‐translational level by promoting proteolytic cleavage by matrix metalloproteinases.^28^ Thus, the expression of each immune‐related molecule is regulated by several stimuli in different ways. We demonstrated that tofacitinib could not block IFNg‐induced decrease of NKG2D ligands in LC‐2/ad. This suggests that IFNγ decreases the expression of NKG2D ligands at the transcriptional level via a pathway other than JAK‐STAT signaling or at the post‐translational level by promoting proteolytic cleavage.

Importantly, an IFNγ‐induced PD‐L1^high^/MHC class I molecules^high^/NKG2D ligands^low^ phenotype is optimal for cancer cells to escape NK cells, since NK cells are inactivated by PD‐L1 and MHC class I molecules, while they are activated by NKG2D ligands on cancer cells. Herein, we showed that the JAK‐STAT inhibitor tofacitinib can block LC‐2/ad cells, thereby changing their characteristic phenotype from being NK cell‐resistant to NK cell‐sensitive by inhibiting IFNγ‐induced reaction, resulting in an enhanced NK cell‐mediated cytotoxicity against IFNγ‐reacted LC‐2/ad cells.

Tumor progression during cancer immunotherapy is the result of immunoselection by host immunity, and our findings suggest possible mechanisms of tumor escape from host immunity during immunotherapy via IFNγ‐induced overexpression of PD‐L1 and MHC class I molecules, and IFNγ‐induced down‐regulation of NKG2D ligands. One major limitation of our current study was that we could evaluate only one NSCLC cell line. Since we have been interested in the comparative analysis between EGF‐ and IFNγ‐induced PD‐L1 expression in NSCLC cell lines, we assessed EGF‐ and IFNγ‐induced PD‐L1 expression in several NSCLC cell lines but found only one cell line, LC‐2/ad, which showed clear enhancement in PD‐L1 expression by both EGF and IFNγ. Since LC‐2/ad, PC‐9, and RERF‐LC‐AI harbor EGFR driver mutation while A549 and RERF‐LC‐KJ are wild type EGFR,^15^ the overcoming effect of tofacitinib in NK cell cytotoxicity in LC‐2/ad is EGFR driver mutation independent.

## CONCLUSIONS

Taken together, our present results suggest that the expression of PD‐L1 could be up‐regulated by several stimuli via different signaling pathways in LC‐2/ad cells. Once cancer cells are attacked by NK cells and T cells, the cancer cells arm IFNγ‐induced PD‐L1 as a “shield” and escape from host immunity. Additionally, IFNγ up‐regulates MHC class I expression and down‐regulates the expression of NKG2D ligands, resulting in a phenotype that can easily escape from NK cells. Our current findings suggest that the JAK‐STAT inhibitor tofacitinib combined with immunotherapy could be a promising strategy as it blocks the immunotherapy‐induced immunoreaction, such as the IFNγ‐induced immunoescape phenotype in NSCLC cells. Although we were concerned about the immunosuppressive effect on tofacitinib against NK cells, less than 3 μM of tofacitinib does not induce any apoptotic effect on NK cells, suggesting that tofacitinib could be a promising agent that can be used in combination with immunotherapy, thereby activating NK cells such as chimeric antigen receptor NK cells, or immunocheckpoint inhibitors targeting KIR to reinforce the NK cell‐mediated immunosurveillance, although it could be adapted to the limited number of NSCLC patients.

## CONFLICT OF INTEREST

Dr. Masao Nakata received research funding from Kyowa Hakko Kirin, Taiho Pharma, Ono Pharma, and Nihon Medi‐Physics Co. The sponsors had no control over the interpretation, writing, and publication of this study. All other authors declare no conflicts of interest.

## ETHICS APPROVAL AND CONSENT TO PARTICIPATE

Our research was approved by the Kawasaki Medical School ethics committee (No. 1217‐5). We did not use any data from patients in this study. Therefore patient consent for publication is not required.

## AUTHOR CONTRIBUTIONS

Riki Okita substantially contributed to the conception, design, acquisition of data, and analysis and interpretation of data. Katsuhiko Shimizu, Shinsuke Saisho, and Yuji Nojima contributed to the interpretation of data and academic input. Riki Okita and Masao Nakata drafted the article and all authors revised it critically for important intellectual content, and agreed to be accountable for all aspects of the work in ensuring that questions related to the accuracy or integrity of any part of the work are appropriately investigated and resolved.

## Supporting information


**Figure S1**. PD‐L1 overexpression is induced by both EGF and IFNγ in LC‐2/ad cells.

**Figure S2**. Expression of PD‐L1 in NSCLC cell lines treated with IFNγ or EGF.

**Figure S3**. Result of human phospho‐RTK array experiments in LC‐2/ad cells.

**Figure S4**. Schematic diagrams summarize how tofacitinib overcomes NK killing in IFNγ‐reacted LC‐2/ad.

## Data Availability

The datasets used and/or analyzed during the current study are available from the corresponding author on reasonable request.
