# Supplementary figures and images for: Tofacitinib overcomes an IFNγ‐induced decrease in NK cell‐mediated cytotoxicity via the regulation of immune‐related molecules in LC‐2/ad
Source: Thorac Cancer. 2021 Jan 24;12(6):775–82. doi: 10.1111/1759-7714.13847 (PMC7952785; doi:10.1111/1759-7714.13847)

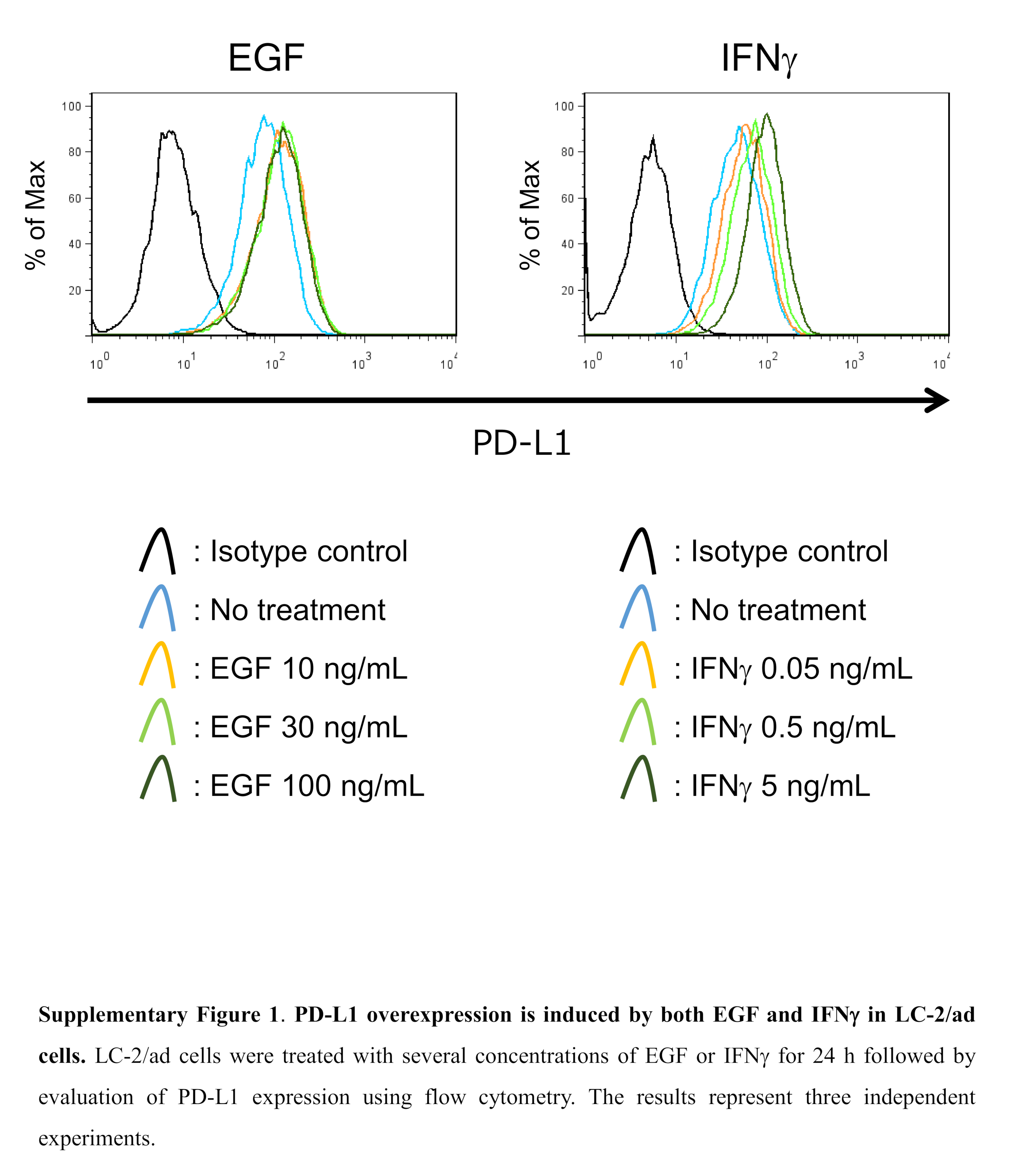

Supplement: Supplementary file 1 — Figure S1. PD‐L1 overexpression is induced by both EGF and IFNγ in LC‐2/ad cells. [file TCA-12-775-s003.tif]

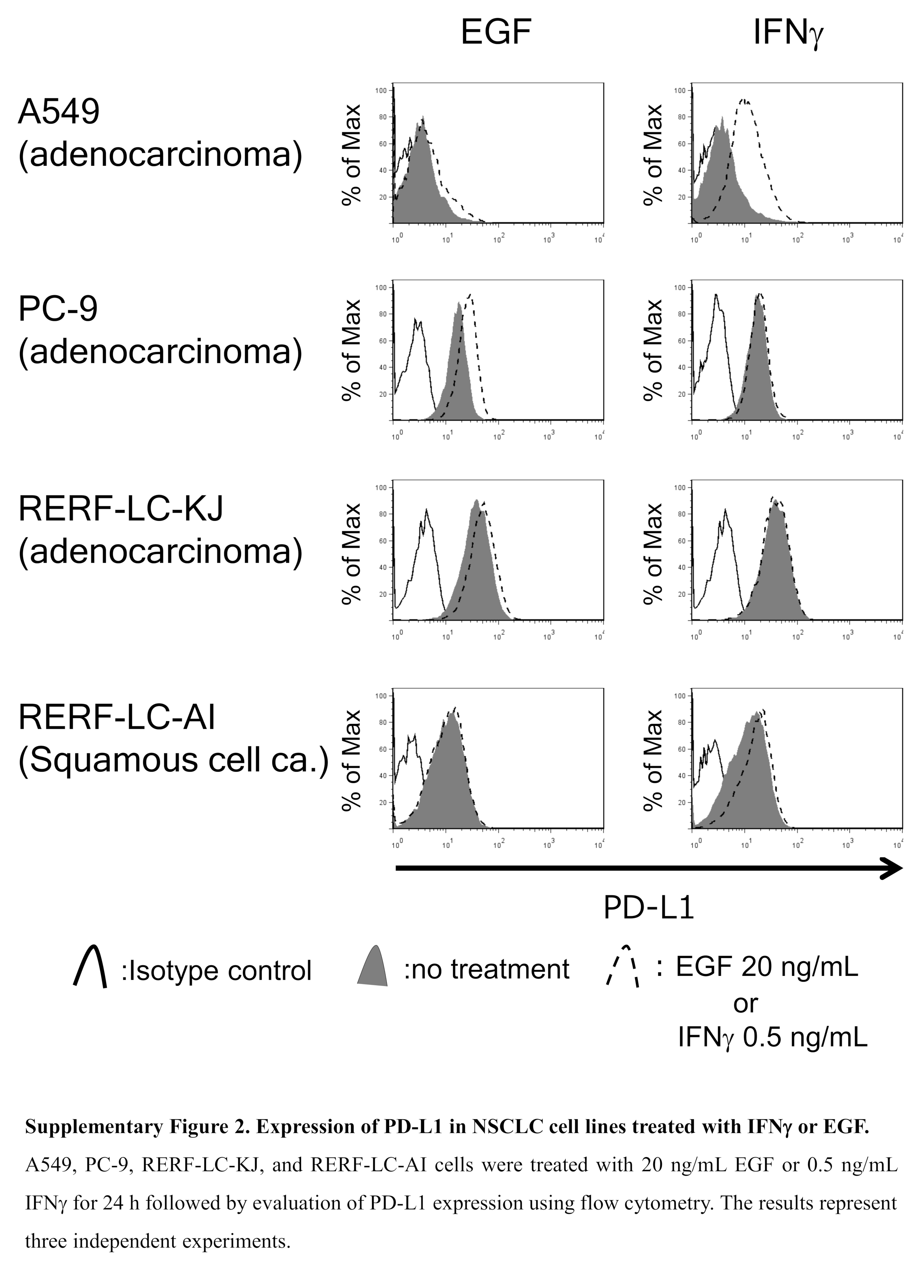

Supplement: Supplementary file 2 — Figure S2. Expression of PD‐L1 in NSCLC cell lines treated with IFNγ or EGF. [file TCA-12-775-s002.tif]

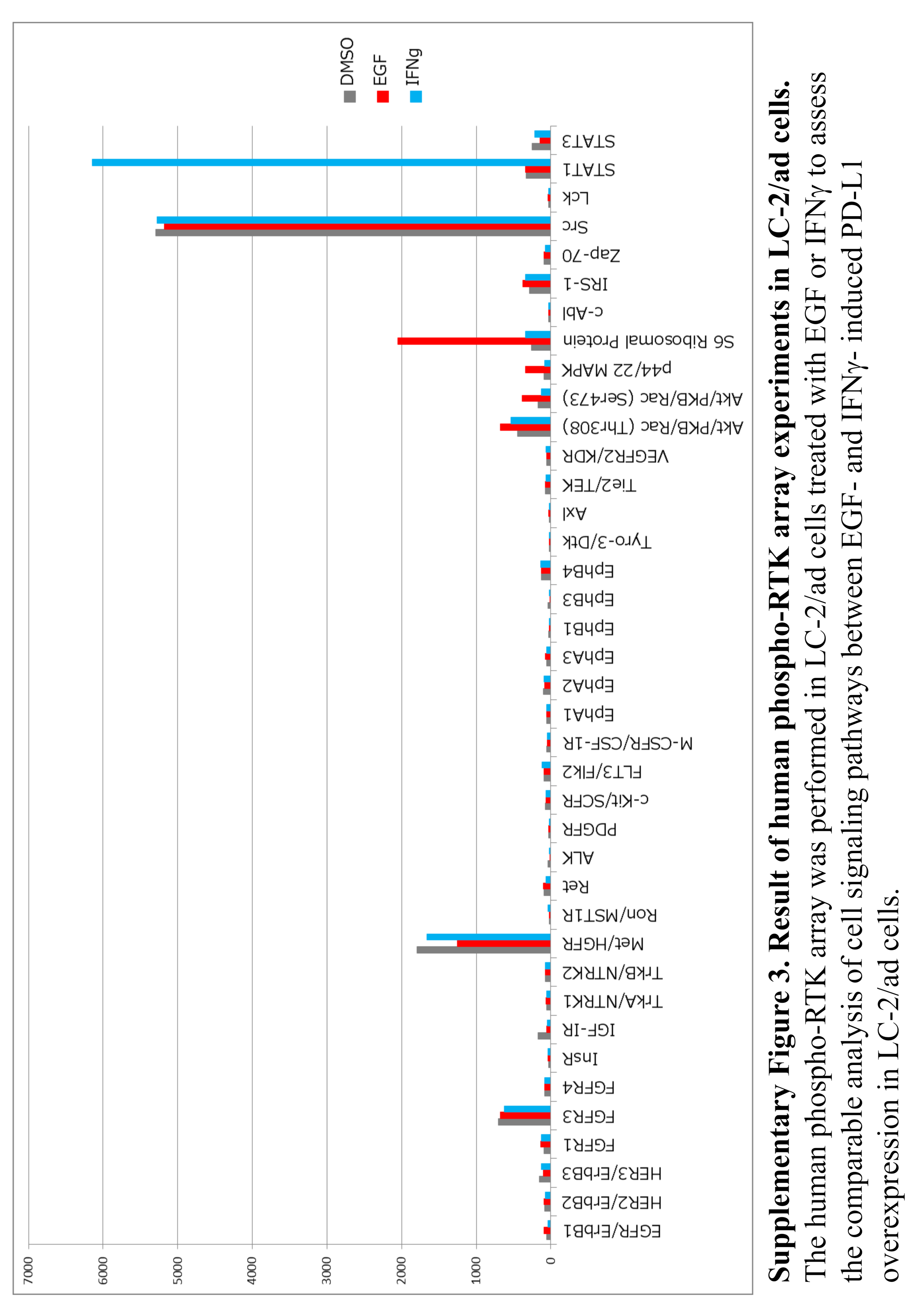

Supplement: Supplementary file 3 — Figure S3. Result of human phospho‐RTK array experiments in LC‐2/ad cells. [file TCA-12-775-s001.tif]

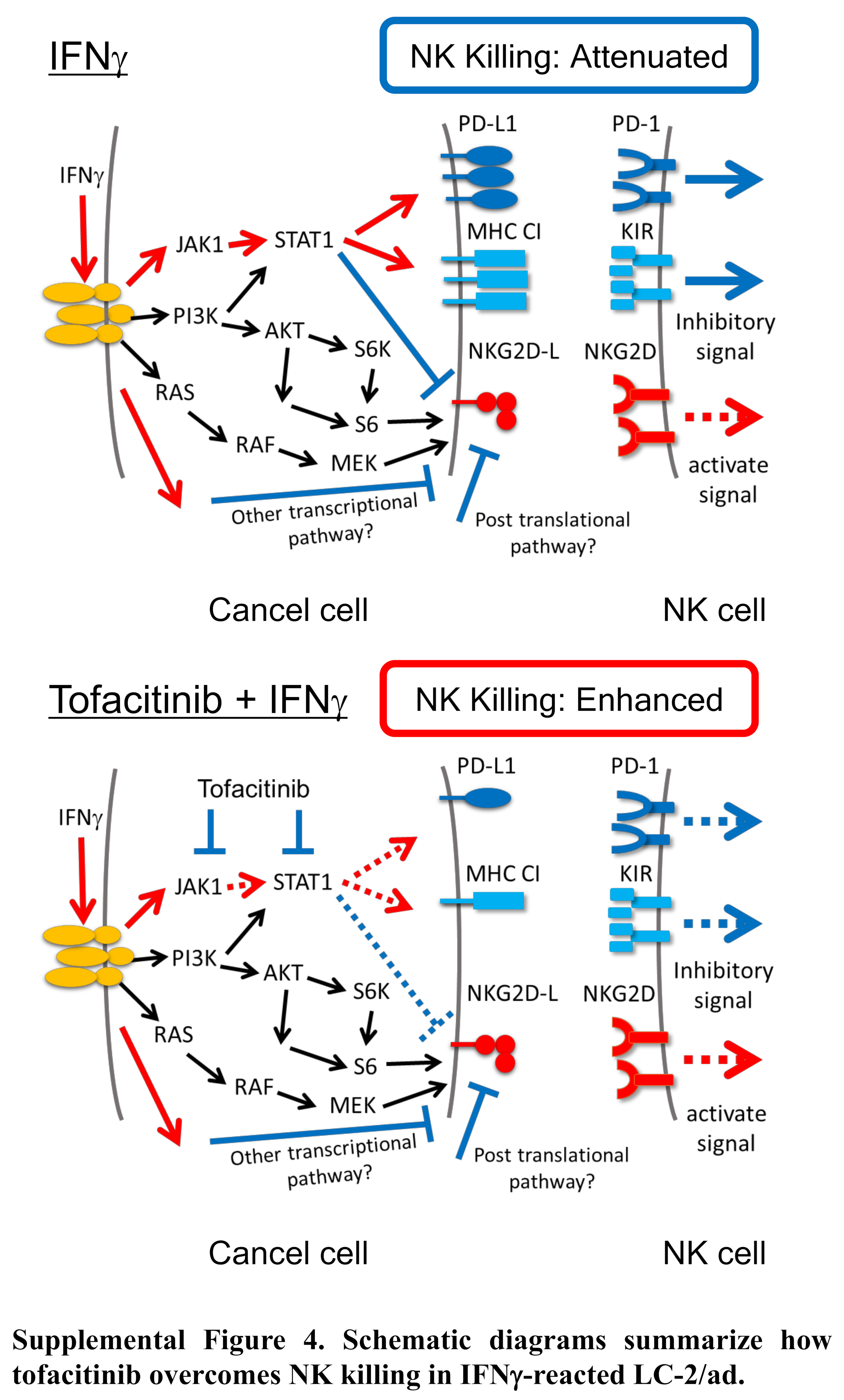

Supplement: Supplementary file 4 — Figure S4. Schematic diagrams summarize how tofacitinib overcomes NK killing in IFNγ‐reacted LC‐2/ad. [file TCA-12-775-s004.tif]
